# Evaluation of Iowa’s anti-bullying law

**DOI:** 10.1186/s40621-016-0080-9

**Published:** 2016-06-07

**Authors:** Marizen Ramirez, Patrick Ten Eyck, Corinne Peek-Asa, Angela Onwuachi-Willig, Joseph E. Cavanaugh

**Affiliations:** 1 Injury Prevention Research Center, The University of Iowa, Iowa City, USA; 2 Departments of Occupational and Environmental Health & Epidemiology, College of Public Health, The University of Iowa, Iowa City, IA USA; 3 Department of Biostatistics, College of Public Health, The University of Iowa, Iowa City, USA; 4 College of Law, University of Iowa, Iowa City, USA

**Keywords:** Violence, School, Law

## Abstract

**Background:**

Bullying is the most common form of youth aggression. Although 49 of all 50 states in the U.S. have an anti-bullying law in place to prevent bullying, little is known about the effectiveness of these laws. Our objective was to measure the effectiveness of Iowa’s anti-bullying law in preventing bullying and improving teacher response to bullying.

**Methods:**

Sixth, 8th, and 11th grade children who completed the 2005, 2008 and 2010 Iowa Youth Survey were included in this study (*n* = 253,000). Students were coded according to exposure to the law: pre-law for 2005 survey data, one year post-law for 2008 data, and three years post-law for 2010 data. The outcome variables were: 1) being bullied (relational, verbal, physical, and cyber) in the last month and 2) extent to which teachers/adults on campus intervened with bullying. Generalized linear mixed models were constructed with random effects.

**Results:**

The odds of being bullied increased from pre-law to one year post-law periods, and then decreased from one year to three years post-law but not below 2005 pre-law levels. This pattern was consistent across all bullying types except cyberbullying. The odds of teacher intervention decreased 11 % (OR = 0.89, 95 % CL = 0.88, 0.90) from 2005 (pre-law) to 2010 (post-law).

**Conclusions:**

Bullying increased immediately after Iowa’s anti-bullying law was passed, possibly due to improved reporting. Reductions in bullying occurred as the law matured. Teacher response did not improve after the passage of the law.

## Background

Bullying, the most prevalent form of violence in schools, is defined as peer-on-peer aggressive behavior that occurs repeatedly over time and arises from a power imbalance (Olweus 1978). Although estimates vary depending on the populations being studied, type of bullying (i.e., relational, verbal, physical or cyber) being assessed and the time periods for recall, recent surveys indicate that bullying occurs in 9–50 % of youth in the United States (Eaton et al. 2012; Pergolizzi et al. 2011; Robers et al. 2014; Wang et al. 2009). Data from 1998 to 2010 suggest that rates are starting to decline (Perlus et al. 2014). However, bullying rates remain highest in middle school, and 3–5 % higher in rural relative to urban school settings (Pergolizzi et al. 2011; Robers et al. 2014; Nansel et al. 2001).

Declines in bullying have been attributed to increased efforts in prevention, including the implementation of state anti-bullying laws (Pergolizzi et al. 2011; Wang et al. 2009; Perlus et al. 2014). Today, all states except for Montana have anti-bullying laws in place, and laws vary in terms of requirements and recommendations. Little is known about the effectiveness of anti-bullying laws on preventing bullying behaviors in youth. A small body of research, mostly qualitative in nature, has focused on the legal content of anti-bullying laws (Stuart-Cassel et al. 2011; U.S. Government Accountability Office 2012; Alley and Limber 2009; Cornell and Limber 2015; Grimm 2012; Limber and Small 2003; Srabstein et al. 2008) and their implementation, but not on behavioral outcomes (Stuart-Cassel et al. 2011; U.S. Government Accountability Office 2012). A recent study compared the effectiveness of different state anti-bullying laws on reducing bullying behaviors among high school students from 25 U.S. states (Hatzenbuehler et al. 2015). That study found that state laws that have a statement of scope, describe prohibited behaviors and require school districts to develop local policies were associated with reduced bullying. While valuable, this cross-sectional study could not assess how behaviors of students and teachers changed after the passage of new laws. This research begins to fill this knowledge gap.

Anti-bullying laws have a theoretical basis, following a socio-ecologic approach in prevention to improve school safety climate through activities at the community-, administration/staff-, and student-levels (Dresler-Hawke and Whitehead 2009; Espelage 2014). Activities addressed in anti-bullying laws - reporting, response strategies, disciplinary action, staffing and training - may occur at various levels of this ecological model (Espelage 2014). Two key individual behavioral outcomes are hypothesized to be impacted by these multi-level activities: (1) acts of student-on-student bullying, and (2) acts of intervention by teachers or adults on campus, due to increased awareness, training and procedures.

The current study – an evaluation of Iowa’s anti-bullying law –tested these two hypotheses by comparing student and teacher behaviors before and after the passage of the law. Iowa Code 280.28 requires schools to adopt an anti-bullying policy that defines acts of bullying, puts into place a process for reporting incidents, and describes consequences and actions for bully perpetrators. With mandates effective in September 2007, Iowa’s schools have had relatively recent experiences in the U.S. in adopting anti-bullying statutes. Furthermore, unlike most states, Iowa has systematically collected student-reported bullying through statewide school-based surveys since 2005, prior to the implementation of the law. Our aim was to evaluate the effectiveness of the Iowa law in reducing bullying and in improving teacher response to bullying incidents. The following hypotheses were tested: 1) immediately after the passing of the law, bullying occurred more often due to increased reporting and the law, if effective, led to decreased bullying after a delayed post-law period, and 2) teachers or adults at school would be more likely to intervene on bullying incidents throughout the post-law period compared with the pre-law period. Findings from this study will strengthen the evidence base for anti-bullying laws.

## Methods

### Participants

Data were drawn from the 2005, 2008 and 2010 Iowa Youth Survey (IYS), a statewide survey of all 6th, 8th and 11th grade students conducted by the Iowa Department of Public Health. All school districts are invited to participate in the survey. About 86 % of public school districts and over 13 % of non-public school districts are represented in the IYS. In school districts that agree to participate, students and their parents may opt out of the survey. Approximately 253,000 students from every county across Iowa participated in the IYS during the study period; this represented about 70 % of 6^th^ and 8^th^ graders and over 61 % of 11^th^ graders in Iowa.

The self-administered questionnaire was given to students by paper-pencil in spring 2005 and online (Survey Monkey) in spring 2008 and 2010. The IYS assesses attitudes and experiences regarding substance use, violence, social competence, and perceptions of the environment. The questionnaire content was determined by a committee of professionals designated by the Iowa Department of Public Health (for additional details regarding the survey and summary reports, see www.iowayouthsurvey.iowa.gov). This research, which utilized de-identified student data, was exempt from Human Subjects Review.

### Measures

Exposure to the anti-bullying law: The main independent variable was exposure to the provisions of Iowa Code 280.28, which by fall 2007 required all school districts to adopt and publicize an anti-bullying policy that defines and prohibits bullying and harassment, and describes procedures for reporting and investigations as well as the consequences of bullying (https://coolice.legis.iowa.gov/Cool-ICE/default.asp?category=billinfo&service=IowaCode&input=280.28). Code 280.28 also encourages primary prevention programming and training (https://coolice.legis.iowa.gov/Cool-ICE/default.asp?category=billinfo&service=IowaCode&input=280.28). To support school districts in the implementation of the law, the Iowa Department of Education and local educational agencies established an informational website; established a state bullying reporting system; and provided trainings throughout the state.

To measure exposure to the law, we categorized students who completed the Iowa Youth Survey (IYS) as follows: student survey data collected in 2005 were categorized as exposed to the pre-law period; student surveys from 2008 as exposed to the one year post-law period; and student surveys from 2010 as exposed to the post-law period.

Bullying: In the IYS, students reported the frequency of being a target of verbal, psychological, physical, or cyberbullying in the past month by answering: “In the last 30-days, how many times have you been bullied at school in the ways listed below:” 1) Verbal bullying: “I was called names, was made fun of, or teased in a hurtful way”; 2) Relational bullying: “Other students left me out of things on purpose, excluded me from their group of friends, completely ignored me, told lies, spread false rumors about me, or tried to make others dislike me”; 3) Physical bullying: “I was hit, kicked, pushed, shoved around, or locked indoors”; and 4) Cyber bullying: “I have received a threatening or hurtful message from another student in an email, on a website, on a cell phone, from pager text messaging, in an internet chat room, or in instant messaging.” Response options were “0 time,” “1 time,” “2 times,” “3–5 times,” “6–10 times’ or “11+ times.” For this analysis, bullying was defined as bullied three or more times in the previous month. This conservative cutpoint was chosen to avoid potential misclassification of being bullied.

Perceived teacher response to bullying (teacher intervention): Students were asked how often teachers or other adults tried to put a stop to bullying using a Likert scale (almost never, once in a while, sometimes, and often/almost always). Teacher response to bullying was analyzed as an outcome impacted by the law in one set of models, and as an explanatory variable (potential covariate) in models examining the impact of the law on being bullied. As an outcome variable, teacher intervention was dichotomized as substantial (often/almost always) and not substantial intervention (almost never/once in a while/sometimes). As an explanatory variable, teacher intervention was treated as an ordinal variable using the entire Likert scale.

School district was included as a random effect in the models to account for dependence of observations within the same school district. While preferable to cluster observations at the school-level, the school variable was not accessible to the research team without permission from each school that participated in the study.

In addition, demographics (grade: 6^th^, 8^th^, 11^th^ grade), gender (male, female), and home living situation (with parents, with grandparents or other relatives, with foster parents, in shelter care, in a residential group or home, or independent living) were considered as potential covariates to explain variability and improve model fit. Selection of grade, gender and living situation was based on a priori knowledge of risk factors for peer-to-peer violence. Other contextual variables (e.g., school connectedness, social support) available from the IYS were considered as possible protective factors in the school environment against bullying; however, conceptually, they function as possible intermediates impacted by law and should not be adjusted for in the models.

### Statistical analysis

Generalized Linear Mixed Models (GLMMs) were used to assess the law’s effect on being bullied and teacher response to bullying. Dichotomous outcomes were modeled for each form of bullying using a binominal distribution and a logic link function. The models using the ordinal outcomes did not lead to appreciably different conclusions than models using binary outcomes. The structure of the GLMM was a mixed effects logistic regression model.

Controlling for grade, gender, ethnicity, and living situation as fixed effects, the models estimated the relative odds of being bullied by comparing pre-law, one year post- and three years post-law time periods. School district was included as a random effect (intercept). The Laplace method, which produces approximate maximum likelihood estimates, was used as a fitting procedure. Separate models were constructed for the different forms of bullying (i.e., verbal, exclusion, physical and cyber). A similar GLMM, also controlling for grade, gender, ethnicity, and living situation, evaluated the impact of the law on teacher intervention, with teacher intervention as the outcome modeled. Possible moderating effects of 1) teacher response and 2) grade on the association between the law and being bullied were also evaluated.

Due to our large sample size, all of the hypothesis tests produced statistically significant *p*-values. As a result, we used the Bayesian Information Criterion (BIC) for our model selection decisions. BIC is often employed in large-sample settings where the modeling objective is descriptive, since it eliminates variables corresponding to negligible effects that are statistically significant merely because of the sample size. The model with the lowest BIC was selected as the candidate model which is rendered most plausible by the data. From the fitted GLMMs, the odds for bullying and intervention were estimated. Statistical analyses were completed using SAS in 2014.

## Results

From pre-law (2005) through post-law (2008, 2010) periods, a total of 253,054 students in the 6th, 8th and 11th grades completed the Iowa Youth Survey, with about 90,000 students surveyed during each year. Students were equally distributed by gender and by grade (about one-third of the sample came from each of the three sampled grades). A total of 84.0 % of the student population was White, and 95.9 % lived at home with their parents.

**Fig. 1:**
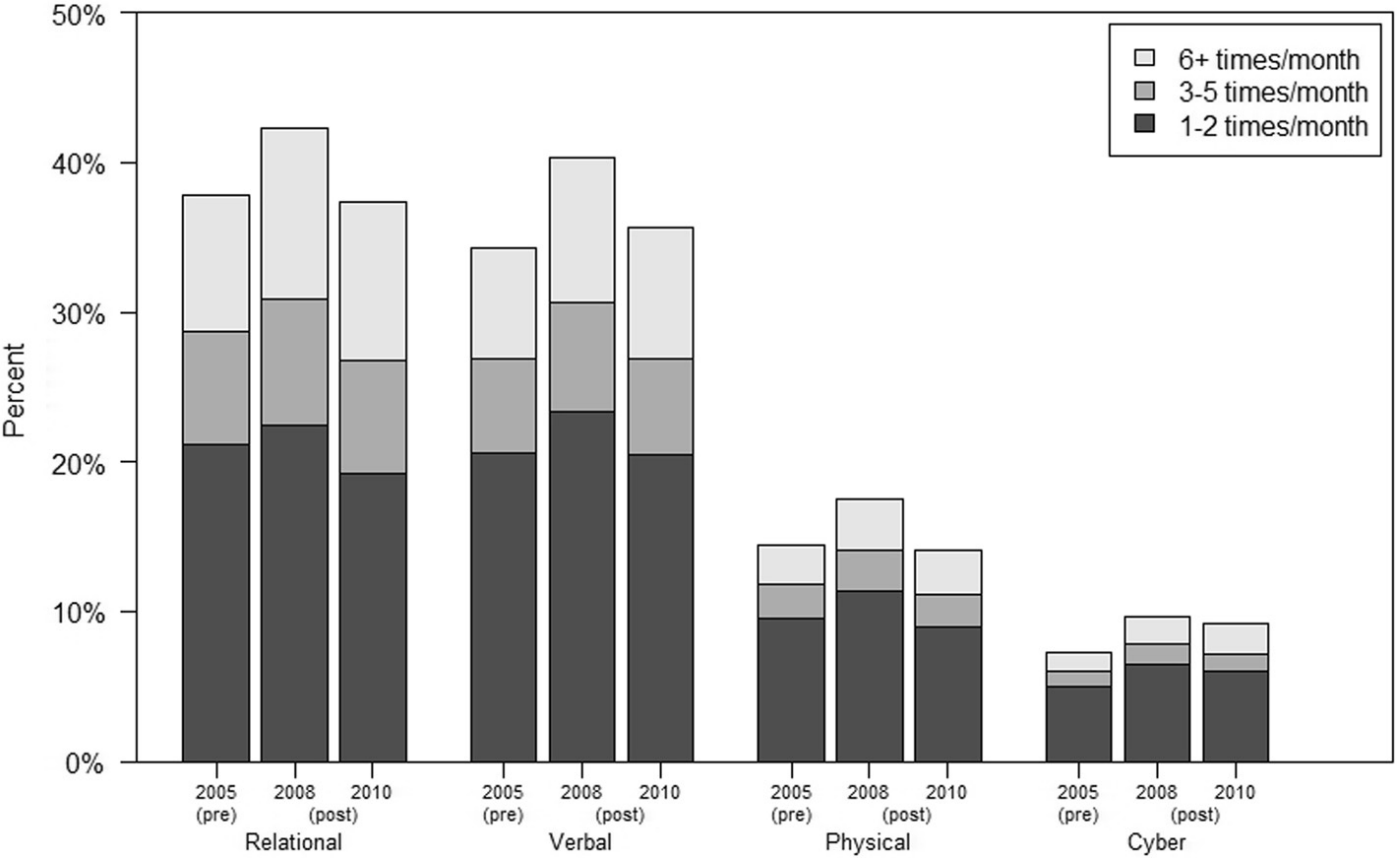
Percent of students who were targets of bullying, Iowa, 2005–2010 (*N* = 248,110)

Over the study period, 47.3 % of students reported being targets of relational, verbal, physical or cyber bullying. Relational and verbal bullying were the most common bullying forms, and they occurred with similar frequency, impacting 38.8 % of students. Physical bullying was a much less common form of bullying and impacted 15.4 % of students. Cyberbullying was the least common form (7–9 % during the study period). For students who had experienced some degree of bullying, over half reported experiencing low levels (1–2 times in the past two months). Compared with pre-law levels in 2005, there was increased relational, verbal and physical bullying in 2008 (one year post-law) followed by a decrease in 2010 (three years post-law) (Fig. 1).

### Impact of the law on bullying

**Fig. 2:**
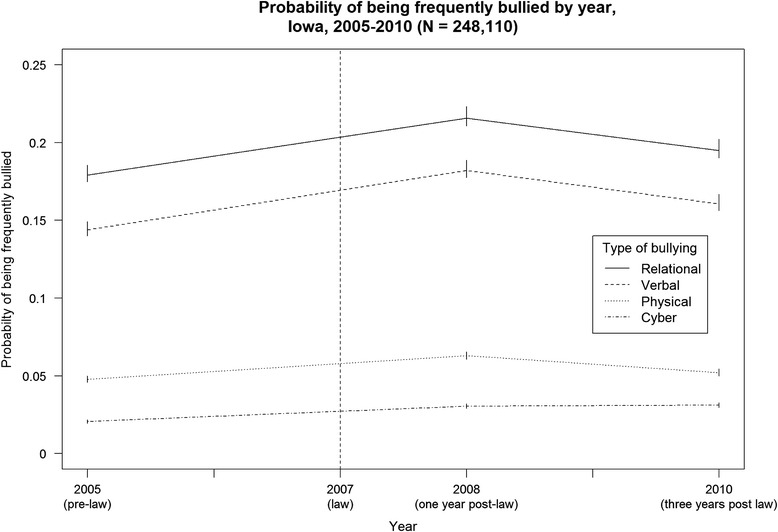
Probability of being bullied by year, Iowa, 2005–2010 (*N* = 248,110)

Each bullying type exhibited an increase in the probability of occurrence from pre-law (2005) to immediate post-law (2008) (Fig. 2). Relational, verbal, and physical bullying each had a decrease in probability of occurrence from one to three years post-law. In contrast, cyberbullying showed a slight increase from 2008 to 2010. Consistent with descriptive statistics, the highest predicted probability was for relational bullying (about 18 % of students), followed by verbal (about 15 %), physical (about 5 %), and cyber (only about 2.5 %). The change over each time period was more pronounced for the more common forms of bullying.

Models adjusted for grade, gender, ethnicity and living situation were consistent with univariable models (Table 1). Across all forms of bullying except cyberbullying, there was an increased odds of being bullied one year post-law followed by a decreased odds three years post-law. However, the odds of being bullied during the three year post-law period still exceeded that of the pre-law period. For example, the odds of relational bullying one year post-law compared to the pre-law period increased by 24 % (OR = 1.24, 95 % CI = 1.21, 1.27) (Model 2, Table 1). However, the odds of relational bullying three years post-law decreased by 13 % compared to one year post law (2008) (OR = 0.87, 95 % CI = 0.85, 0.90). Compared to pre-law periods, however, the odds of relational bullying the third year after the law remained slightly higher (OR = 1.08, CL% = 1.05, 1.11).

**Table 1:** Odds ratios for relational, verbal, physical and cyber bullying victimization by exposure to the law and by teacher intervention

Bullying type	Exposure to law	Model 1	Model 2
		OR (95 % CI)	OR (95 % CI)
Relational	1 year post-law vs. Pre-law	1.27 (1.24, 1.30)	1.24 (1.21, 1.27)
	3 years post-law vs. Pre-law	1.14 (1.11, 1.17)	1.08 (1.05, 1.11)
	3 years post-law vs. 1 year post-law	0.90 (0.88, 0.92)	0.87 (0.85, 0.90)
	Teacher intervention vs. No intervention		0.65 (0.64, 0.65)
Verbal	1 year post-law vs. Pre-law	1.32 (1.29, 1.36)	1.29 (1.26, 1.33)
	3 years post-law vs. Pre-law	1.15 (1.12, 1.19)	1.10 (1.06, 1.13)
	3 years post-law vs. 1 year post-law	0.87 (0.85,0.90)	0.85 (0.82, 0.87)
	Teacher intervention vs. No intervention		0.64 (0.64, 0.65)
Physical	1 year post-law vs. Pre-law	1.32 (1.26, 1.38)	1.28 (1.22, 1.33)
	3 years post-law vs. Pre-law	1.09 (1.04, 1.15)	1.03 (0.98, 1.08)
	3 years post-law vs. 1 year post-law	0.83 (0.79, 0.87)	0.81 (0.77, 0.84)
	Teacher intervention vs. No intervention		0.61 (0.60, 0.62)
Cyber	1 year post-law vs. Pre-law	1.49 (1.40, 1.58)	1.44 (1.36, 1.53)
	3 years post-law vs. Pre-law	1.57 (1.48, 1.68)	1.47 (1.38, 1.57)
	3 years post-law vs. 1 year post-law	1.06 (1.00, 1.12)	1.02 (0.96, 1.08)
	Teacher intervention vs. No intervention		0.55 (0.54, 0.56)

Model 1: Generalized Linear Mixed Models clustering on school district

Model 2: Generalized Linear Mixed Models clustering on school district and controlled for grade, gender, ethnicity, and living situation

Notably, however, the law did not appear to impact cyberbullying to the same extent. While we observed an increased odds of cyberbullying in 2008 (one year post-law) (OR = 1.44, 95 % CL = 1.36, 1.53), we did not see a decrease in 2010 (three years post-law) but rather a comparable odds, as reflected by a slight although not statistically significant increase compared to one year post-law (OR = 1.02, 95 % CL = 0.96, 1.08)

### Impact of the law on teacher intervention

**Fig. 3:**
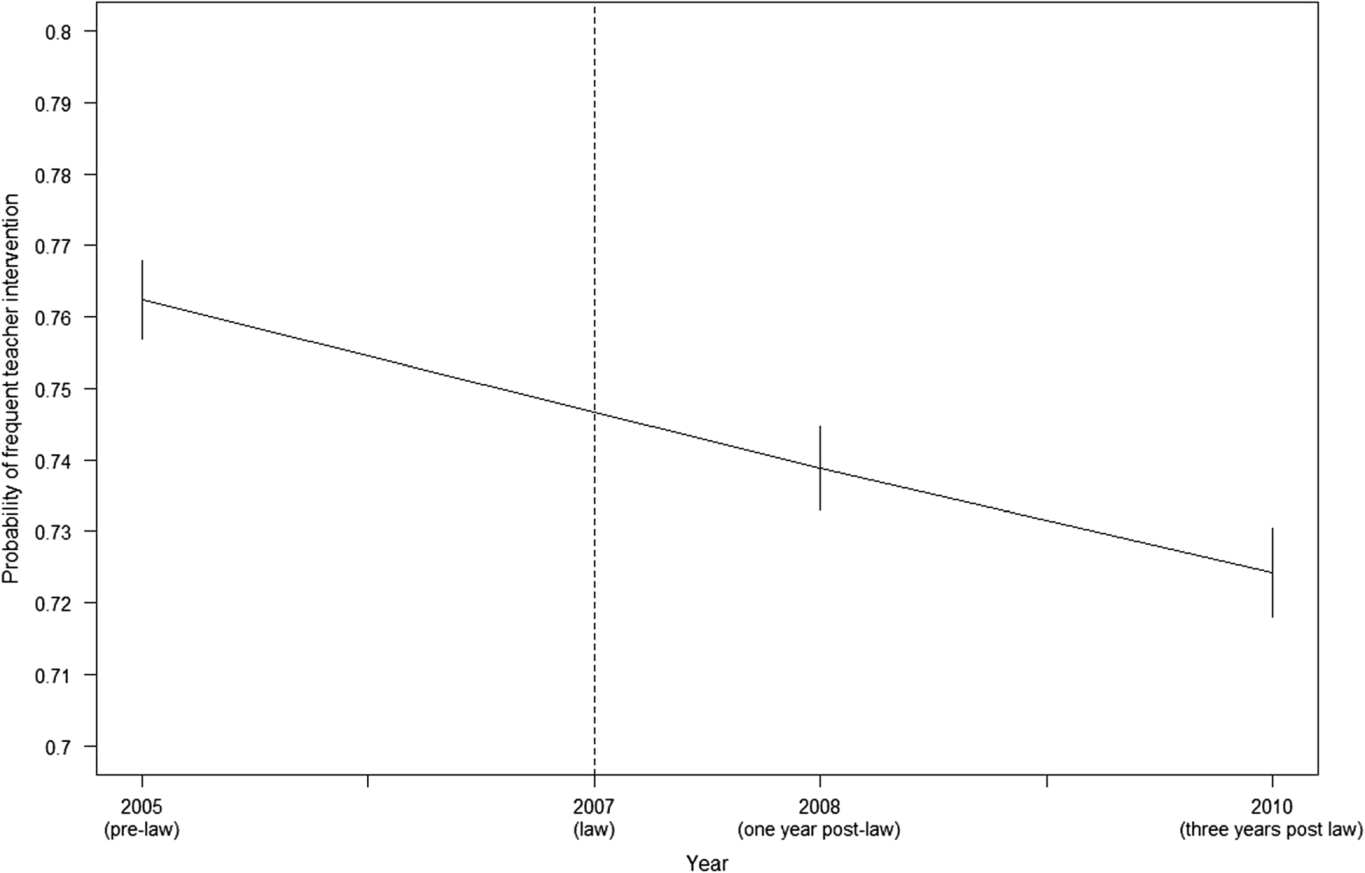
Probability of teacher intervention by year, Iowa, 2005–2010 (*N* = 250,941)

For all years, teacher intervention was common: more than 70 % of students reported that teachers intervened often or very often. Teacher intervention showed a decrease from pre-law (2005) (probability = 0.76) to one year post-law (2008) (probability = 0.74), and from pre-law to three years post-law (2010) (probability = 0.73) (Fig. 3).

In models with teacher intervention as an outcome variable adjusted for grade, gender, ethnicity, and living situation, there was a reduced odds of teacher intervention one year post-law compared with the pre-law period (OR = 0.89, 95 % CL = 0.88, 0.90).

### Testing for the interaction effects of teacher intervention and grade with the law

In the mixed effects logistic regression models for bullying, no evidence of an interaction effect of teacher intervention and law was detected based on the BIC statistic. However, a grade and law interaction was found for relational and verbal bullying. Effect estimates were not appreciably different across grades and followed a similar trend. The only exception was seen for 11^th^ graders, whereby the odds of verbal bullying at three years post-law was 14 % lower than levels from the pre-law period (OR = 0.86, 95 % CL = 0.78, 0.94).

A strong relationship between teacher intervention and bullying was found (Estimates from Model 2, Table 1, not shown). Across all forms of bullying, increasing teacher intervention one unit on the ordinal Likert scale (e.g., from once in a while to sometimes, from sometimes to often) was associated with 35 to 46 % decreased odds of bullying. The general trends for other confounding variables were as follows: 8^th^ and 11^th^ graders both had lower odds of relational, verbal and physical bullying than 6^th^ graders. However, the odds of cyber bullying was 30 % greater for 8^th^ and 11^th^ graders than 6^th^ graders. African American and Native American students also had increased odds of verbal, physical and cyber-bullying than white students. Living with parents was protective against all forms of bullying.

## Discussion

This study contributes to a small body of research evaluating the effectiveness of anti-bullying laws on individual behaviors. Our findings provide support for the socioecological approach to prevention, which purports that community-level strategies (i.e., policies and laws) have a trickle-down effect. Anti-bullying laws instituted at the higher levels of the social ecology (state or community) are associated with changes in student bullying behaviors as well as teacher response to bullying. Granted, the specific mechanisms through which this occurs are largely unknown and cannot be gleaned from this current study. For example, we know little about the kinds of prevention activities or statutes mandated by the law and implemented in communities and schools that lead to improved teacher response or reduced bullying perpetration. However, this study does points us to those next steps for research. Several notable findings from our research are described in more detail.

### Impact of the law on student bullying behaviors

Findings indicated an increase in reported bullying behavior in the year immediately following implementation of the law, and then an encouraging decrease in the odds of being bullied in 2010 (three years post-law) compared with 2008 (one year post-law). This pattern was observed for all types of bullying except cyberbulling, which increased throughout the study period. Also notable was a slightly different trend found in 11^th^ graders, who had an increased odds of verbal bullying one year post-law followed by a significant decrease in the odds of bullying to levels below pre-law levels. A recent national study found national bullying rates in the US to decrease from 2005 to 2010, which in fact opposes our states’ experience of an increase from 2005 to 2008 (Perlus et al. 2014).

The lack of a control group (i.e., another state for comparison) in this study limits our ability to fully attribute the patterns of bullying to the law. The increase in Iowa’s bullying rates from 2005 to 2008, however, reflects influences specific to Iowa, and one interpretation is that the law led to increased awareness and thereby reporting. Likely, awareness naturally occurred as an immediate output of the law: the passage of the law attracted media attention, and the Iowa Department of Education established a number of initiatives to enhance knowledge of the state statutes. A similar post-law effect, perhaps due to increased awareness, was observed in another study that evaluated the immediate effect of Maryland’s ban of “Saturday night special” handguns (Webster et al. 2002).

The reduction in bullying rates from 2008 to 2010 could, on the other hand, be attributed to influences beyond Iowa’s law, such as other anti-bullying efforts at the national or international level or even a mixture of state, national or international efforts. As suggested by Perlus et al. (2014), reductions in bullying rates found across the country from 2005 to 2010 may reflect bullying prevention efforts and campaigns at the national and international level independent of the law (Perlus et al. 2014). The finding of a significant decrease in bullying in 2010 among 11^th^ graders to levels below 2005 is notable. This suggests that perhaps the law – which emphasizes reporting, investigations and disciplinary actions - has greater immediate impact in high schools. It is unclear if these types of strategies work better for high school than middle school students, or if high schools are doing better in implementing the statutes of the law. Further research is needed to understand this phenomenon, and to furthermore explore intervention strategies for middle school when prevalence of bullying is highest.

Disentangling the effects of Iowa’s law from other efforts is not so easy because of difficulties in finding a comparison state without an anti-bullying law and with comparable bullying data. At present, all but one state (Montana) has an anti-bullying law. More importantly, data on bullying experiences were not collected routinely from states until 2011 by the Centers for Disease Control and Prevention (CDC) Youth Behavioral Risk Surveillance System, and even since then, CDC collects national bullying data only at the high school level (Eaton et al. 2012). Another data source, the Child Health Behavior Survey, includes international data on bullying behaviors, but does not include a state-based sample in the U.S. (Pergolizzi et al. 2011; Wang et al. 2009; Perlus et al. 2014). Hence, our research uses the best available data source to study behavior change among middle school students before and after the passage of an anti-bullying law in middle schools.

The increase in cyberbullying in the presence of law is noteworthy. On one hand, if unbiased, results suggest that Iowa’s law may confer more protection against traditional forms of face-to-face bullying. Cyberbullying is likely the most difficult form of bullying for schools to address, with constant technological advancements (e.g., new social networking sites, new types of devices with online accessibility like iPADs, and online gaming sites) and increased access among youth (Madden et al. 2013). This explosion in internet accessibility leads to new avenues for cyberbullying and increased exposure (Wang et al. 2009; Bailin et al. 2014; Kowalski and Limber 2007). Perhaps, anti-bullying laws have not kept up with technological advancements, and this gap may explain why the prevalence of cyberbullying was not influenced by the presence of Iowa’s anti-bullying law. On the other hand, these findings may not represent the law’s true impact on cyberbullying. We were unable to measure exposure to internet accessibility which is exponentially increasing. Without accounting for exposure, effect estimates may be biased especially since the baseline prevalence of cyberbullying is quite low (below 9 %). Future studies are therefore needed to disentangle impacts of the law on cyberbullying in a manner that exposure to technology is accounted.

### Impact of the law on teacher intervention

Our findings have practical implications for the field and call attention to one of the *recommended* but not required elements of Iowa’s law - “Develop a process to provide school employees, volunteers, and students with skills and knowledge to help reduce incidents of bullying.” According to our study, teacher intervention was associated with almost a 50 % reduction in the odds of bullying. Unfortunately, the law did not improve teacher intervention over time. As one interpretation, increased awareness among students (who reported on teacher intervention) may have increased expectations of teacher intervention and differential reporting post-law. Another interpretation is that the policy did not truly impact the extent to which teachers or adults at school intervened on bullying. Although disappointing, our finding points to a need for focused intervention training of teachers and adults on campus, a statute not found in many state anti-bullying laws.

### Limitations

Aside from the lack of a control state, this study has other limitations. Although the Iowa Department of Education provided support to schools to implement the provisions of the anti-bullying law, the specific activities that districts and schools have engaged in after the passage of the law are not yet known. While research, including our ongoing studies in Iowa, has begun to evaluate how schools implement requirements of anti-bullying laws (Stuart-Cassel et al. 2011; U.S. Government Accountability Office 2012), studies are needed to examine how implementation modifies the impact of the law. The Iowa Youth Survey, which captures data by self-report, is prone to response bias, and differences in survey administration may lead to some level of reporting bias. By using bullying three or more times in the past month as the outcome of interest, we reduce potential over-reporting. However, if response bias is present, it is likely non-differential since the survey was administered independently of the implementation of the law.

## Conclusions

This research has important implications for policy makers, public health and education. Our study suggests that laws might impact relational, verbal and physical bullying, through first an immediate effect on increased awareness and increased reporting and then a trend towards longer-term effects on improved (reduced) prevalence. The law did not impact the extent to which teachers intervened on bullying incidents as school, however. These findings underscore the need to focus efforts on developing and improving policies that target bullying behaviors of students and intervention activities of teachers. Continued research is also needed to identify the most effective policies.

In particular, studies are needed to measure the kinds of prevention activities that are being implemented on the ground by schools in response to anti-bullying statutes, to identify the statutes that impact specific behaviors, and to assess the long-term effects of the law after saturation of prevention activities. Also needed are nuanced studies to examine the impact of laws and policies on cyberbullying, accounting for exposure to technology.
